# Fate of Allochthonous Dissolved Organic Carbon in Lakes: A Quantitative Approach

**DOI:** 10.1371/journal.pone.0021884

**Published:** 2011-07-14

**Authors:** Paul C. Hanson, David P. Hamilton, Emily H. Stanley, Nicholas Preston, Owen C. Langman, Emily L. Kara

**Affiliations:** 1 Center for Limnology, University of Wisconsin, Madison, Wisconsin, United States of America; 2 Department of Biological Sciences, Waikato University, Hamilton, New Zealand; 3 Civil and Environmental Engineering, Bacteriology, University of Wisconsin, Madison, Wisconsin, United States of America; US Dept. of Agriculture – Agricultural Research Service (USDA-ARS), United States of America

## Abstract

Inputs of dissolved organic carbon (DOC) to lakes derived from the surrounding landscape can be stored, mineralized or passed to downstream ecosystems. The balance among these OC fates depends on a suite of physical, chemical, and biological processes within the lake, as well as the degree of recalcintrance of the allochthonous DOC load. The relative importance of these processes has not been well quantified due to the complex nature of lakes, as well as challenges in scaling DOC degradation experiments under controlled conditions to the whole lake scale. We used a coupled hydrodynamic-water quality model to simulate broad ranges in lake area and DOC, two characteristics important to processing allochthonous carbon through their influences on lake temperature, mixing depth and hydrology. We calibrated the model to four lakes from the North Temperate Lakes Long Term Ecological Research site, and simulated an additional 12 ‘hypothetical’ lakes to fill the gradients in lake size and DOC concentration. For each lake, we tested several mineralization rates (range: 0.001 d^−1^ to 0.010 d^−1^) representative of the range found in the literature. We found that mineralization rates at the ecosystem scale were roughly half the values from laboratory experiments, due to relatively cool water temperatures and other lake-specific factors that influence water temperature and hydrologic residence time. Results from simulations indicated that the fate of allochthonous DOC was controlled primarily by the mineralization rate and the hydrologic residence time. Lakes with residence times <1 year exported approximately 60% of the DOC, whereas lakes with residence times >6 years mineralized approximately 60% of the DOC. DOC fate in lakes can be determined with a few relatively easily measured factors, such as lake morphometry, residence time, and temperature, assuming we know the recalcitrance of the DOC.

## Introduction

At the global scale, lakes number more than 300 million [1] and may have significant effects on regional carbon balances [2], [3]. They act as vents to the atmosphere for inorganic carbon accumulated in ground and surface waters and as storage and mineralization sites for organic material derived from terrestrial production [2], [4]. In turn, terrestrial carbon affects lake properties, including water color, thermal stability, water chemistry, community composition, and higher trophic levels [5]–[8]. Despite the abundance of information on effects of organic carbon on several lake attributes, we know surprisingly little about the relationship between terrestrial inputs and lake responses over time scales of ecological significance, from days to weeks to seasons.

Understanding the roles lakes play in landscape carbon budgets requires that we quantify the magnitude and degradability (or recalcitrance) of the organic carbon (OC) fluxes and that we understand how lakes process those carbon loads [9]. Both aspects are challenging. For many lakes, measuring the loads is difficult because inputs are diffuse and highly variable through time. Furthermore, the recalcitrance of the load, especially in terms of the biological availability of the OC, is very difficult to quantify. Thus, we have three components that can have high uncertainty: the magnitude of the load, the recalcitrance of the load, and the processing capacity of the lake.

To reduce the complexity of these issues, it can be helpful to focus on the most abundant fraction of the organic pool, dissolved organic carbon (DOC) [10]. Even if we have highly uncertain estimates of the allochthonous contributions to lake DOC, we can attribute changes in observed lake DOC to multiple sources and sinks by making some simple assumptions. To illustrate, we provide this simplified mass balance model of organic carbon processing rates in lakes:
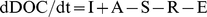
(1)where *I* is allochthonous input, *A* is autochthonous contribution, *S* is sedimentation, *R* is mineralization (respiration plus photo-oxidation), and *E* is export. If we assume that *S* of DOC is negligible, but see [11], that *A* can be estimated from primary productivity [10], [12], and that *E* can be calculated from hydrologic outflow and lake DOC concentration, then only *I* and *R* remain. Unfortunately, these terms are directly related in equation 1 and are not mathematically separable when both are unknown. In cases in which we wish to estimate *I*, for example, and we assume we know *R* from literature values, uncertainty in *R* translates directly to uncertainty in *I*. If by chance mineralization is over-estimated, then the load will be over-estimated as well. Thus, reducing our uncertainty about *R* will help us solve for *I*, which in turn is necessary in determining the role lakes play in the landscape-scale carbon budget.

Respiration at the ecosystem scale (*R_E_*) is particularly challenging to estimate. Scaling measurements of respiration made under controlled laboratory conditions (*R_0_*) [13]–[15] are riddled with challenges associated with the spatio-temporal heterogeneity in temperature, light, and oxic conditions in lakes. In studies that have estimated R at the ecosystem scale [7], [12], [16], little attention has been placed on how the recalcitrant nature of the DOC or the nature of the lake affect scaling, i.e., how we derive *R_E_* from *R_0_*. Critical components of the scaling are the temperature, oxygen, and light environment to which the DOC is exposed. These can be highly variable in space and time, and may be controlled by lake characteristics, such as lake size, hydrologic residence time, and water clarity.

How does DOC recalcitrance, in combination with the processing capacity of lakes, control the fate of DOC loads to lakes? We address this question using a one-dimensional hydrodynamic-water quality model, calibrated to data from the North Temperate Lakes Long Term Ecological Research (NTL LTER) program. We simulate lakes over orthogonal gradients of lake size, trophic state, and recalcitrance of loads to study the relative importance of these factors for determining DOC fates. We found that a few lake characteristics related to size and trophic state were important in determining DOC fate. Equally important, though perhaps more uncertain, is the recalcitrant quality of the DOC load.

## Results

### Model calibration

**Figure 1:**
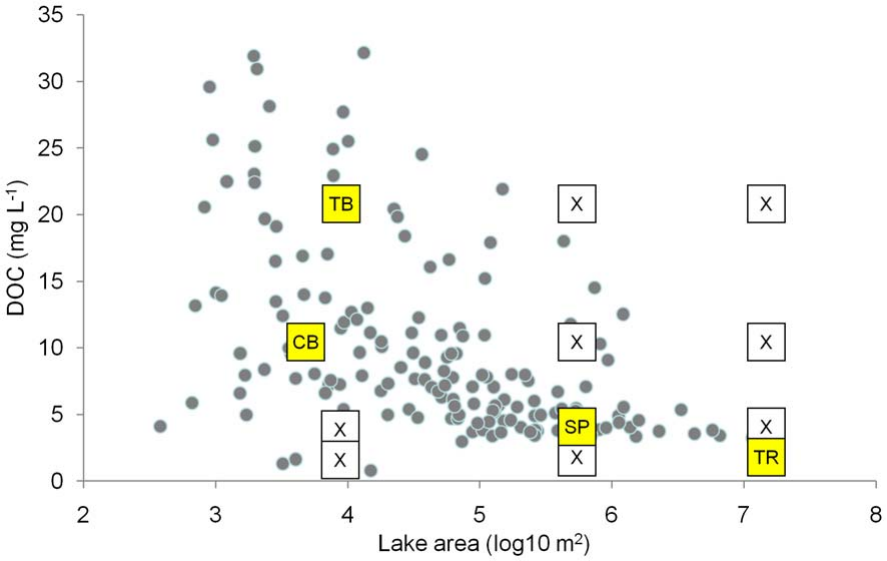
Calibration lakes embedded in points from a survey of lakes representing the NHLD. For each of the 16 lakes, represented by boxes, three different load recalcitrant values (i.e., *R_0_*) were simulated. The yellow boxes are the calibration lakes. Total simulations = 48. Dots are taken from Hanson et al. [19].

**Figure 2:**
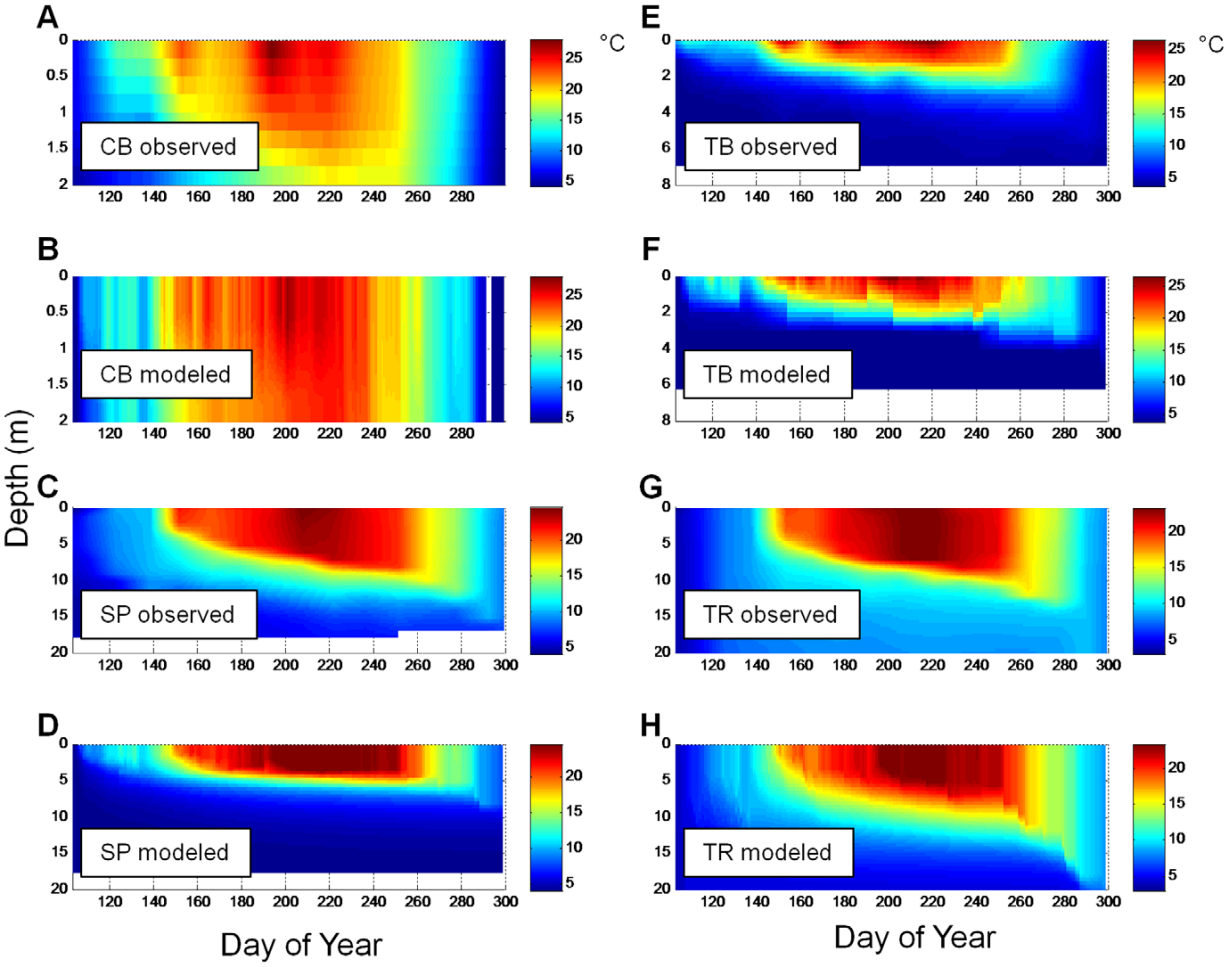
Observed and modeled temperature profiles through the open water season for the calibration lakes. All calibration simulations use *R_0_* = 0.005 d^−1^.

**Figure 3:**
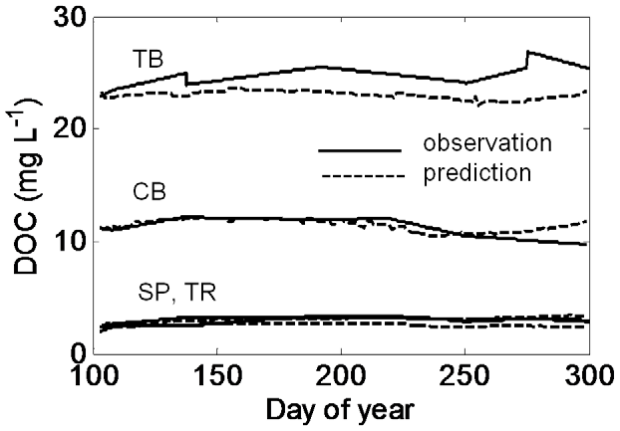
Observed and modeled whole-lake mean DOC concentrations. All simulations are for values of *R_0_* = 0.005 d^−1^. Values are hypsometrically weighted mean water column values.

Results from the model calibration process show predictions approximated to observations for the four calibration lakes, which covered broad ranges in lake area and DOC concentration (Fig. 1). All lakes showed seasonal stratification, although CB showed some short periods of near isothermal conditions (Fig. 2). TB, which is a small lake with high DOC concentration, had the shallowest mixed layer and the coolest depth-integrated temperature over the simulation. Predicted and observed temperatures were strongly correlated (*r*): CB (0.93); SP (0.87); TB (0.94); and TR (0.94). As a collection, the lakes show broad ranges in their thermal properties. Overall, we met the goal of reproducing what we consider to be the important lake attributes in this study – thermal properties and mixing regimes. The mean observed lake DOC concentrations were well represented, even though the details of subseasonal dynamics were not (Fig. 3). An exception was TB, in which DOC predictions were slightly lower than observations. Considering the approach was to fix the daily loads to eliminate them as a confounding factor in the remainder of the analysis, the agreement between observations and predictions was encouraging. One of the purposes of calibration was to determine the DOC load required to reproduce the observed lake concentrations, given the assumed value of DOC respiration (*R_0_*) of 0.005 d^−1^. Under these conditions, the annual areal loads required to produce near constant DOC for each lake were 60 (TR), 55 (SP), 50 (CB), and 190 (TB) g C m^−2^ y^−1^, respectively. It is important to note that these loads, determined through calibration, are strongly influenced by our assumed model parameters and hydrologic residence times.

**Figure 4:**
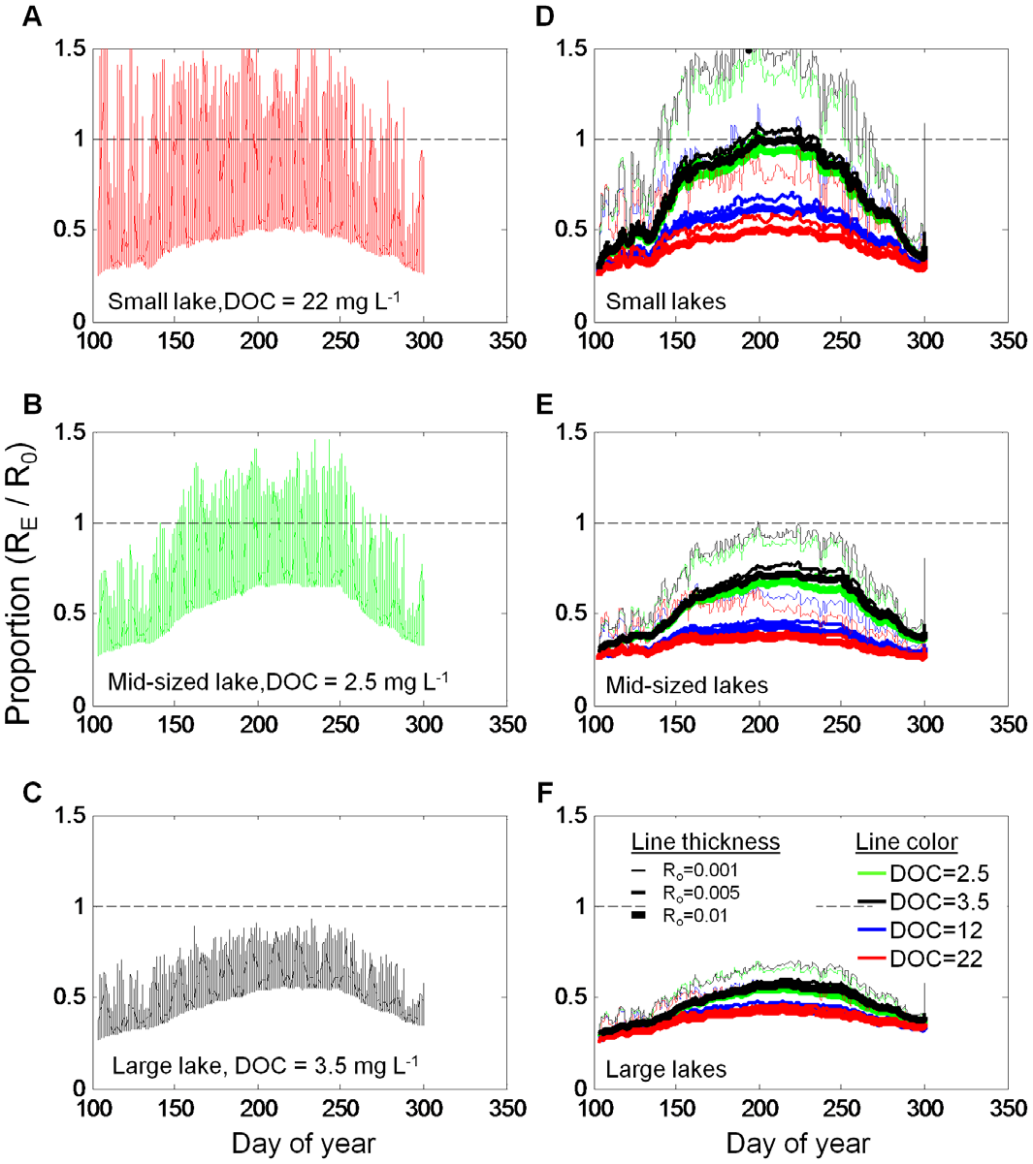
Comparison of *R_E_* and *R_0_* for three different lake sizes. (A–C) Hourly *R_E_* as a proportion of *R_0_* under nominal conditions for the three calibration lakes. (D–F) Smoothed daily *R_E_* as a proportion of *R_0_* under all conditions. Line color represents DOC condition and line thickness represents *R_0_* values.

Ecosystem R (*R_E_*) varied markedly within and between simulations. In the four calibration lakes, *R_E_* was highly variable through time for each lake, but in ways that were lake specific (Fig. 4 A–C). Under calibrated conditions, *R_E_* varied most in TB, with the *R_E_*∶ *R_0_* ratio exceeding one on many days. Conversely, in TR, *R_E_*∶ *R_0_* never exceeded one. When results from all simulations are plotted (Fig. 4D–F, note that data are smoothed), lake area, DOC concentration, and even *R_0_* affect the *R_E_*∶ *R_0_* ratio. The ratio was highest in the smaller lakes (Fig. 4D) with the lowest DOC. Curiously, *R_E_* was similarly low across lakes when DOC was low and *R_0_* was high. In the large lakes (Fig. 4F), none of the scenarios produced high *R_E_*∶ *R_0_*.

**Figure 5:**
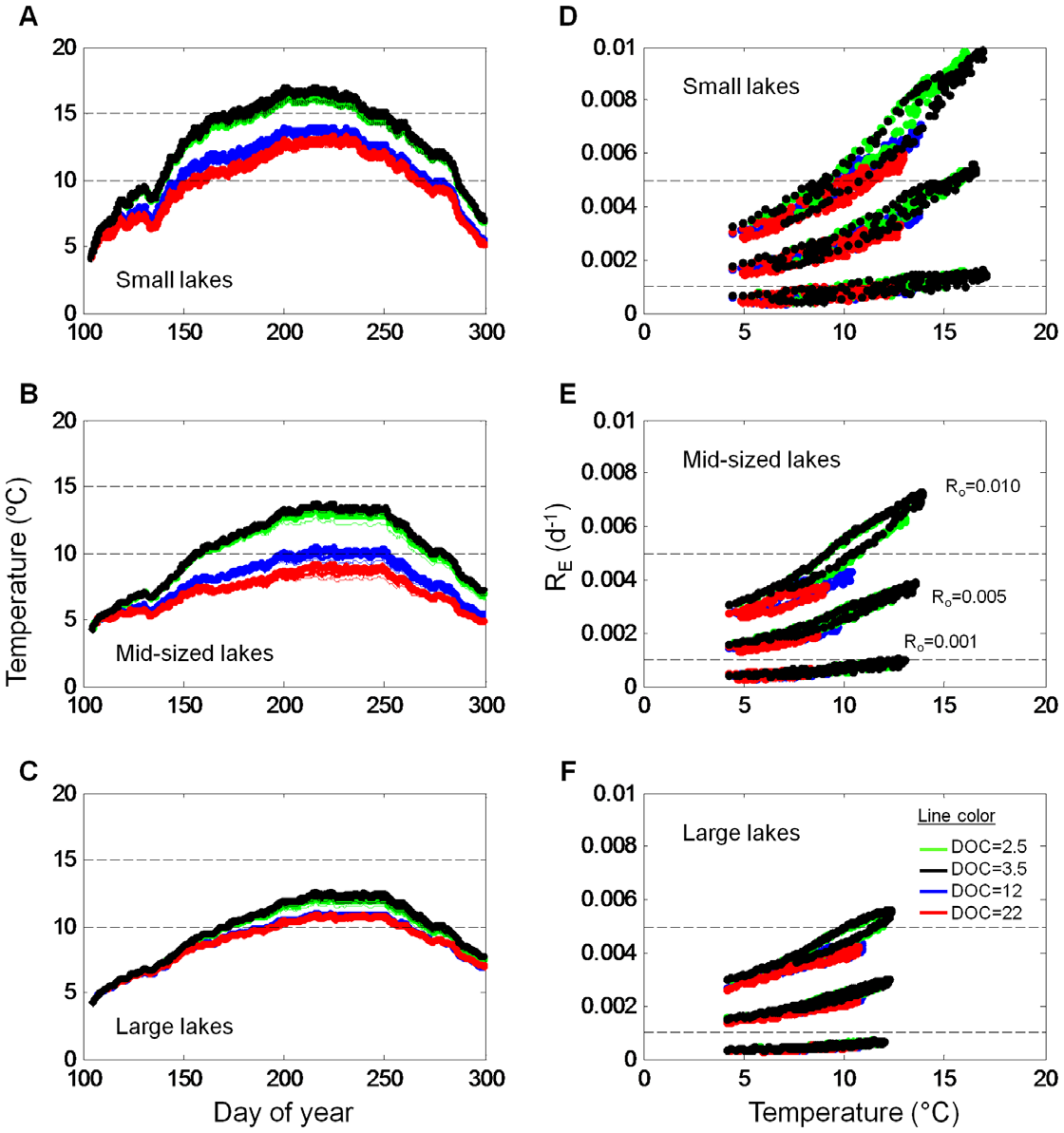
The relationship between lake temperature and *R_E_*. (A–C) Mean lake temperature through time. Each lake has four different DOC conditions. Dashed lines are at 10°C and 15°C to allow for easier visual comparison among panels. (D–F) *R_E_* versus temperature. Each lake has three different *R_0_*, which tend to cluster together, and within each cluster are four different DOC conditions. Dashed lines are at *R_0_* values. Lake sizes are the same as in Fig. 4.

Seasonal changes in mean lake temperature exerted strong influence on *R_E_*. Seasonal variation in water temperature differed among lake areas and among DOC concentrations within lake areas (Fig. 5A–C). The largest seasonal change was in the smaller lakes, while the smallest change was in the largest lake. Biggest differences among simulations within a given lake area occurred in the smaller and midsized lakes (Fig. 5A, B, respectively). The scenario resulting in highest water temperatures was is in the small lake when DOC concentration was low. The general trend is an inverse relationship between DOC and water temperature.

When all data from all scenarios are plotted, *R_E_* shows a direct and nonlinear change with temperature (Fig. 5 D–F). In each panel in Fig. 5, the upper cluster of points corresponds to *R_0_* = 0.010 d^−1^, the middle to *R_0_* = 0.005 d^−1^ and the bottom to *R_0_* = 0.001 d^−1^. The different colors of dots represent the different simulated DOC conditions. In the small, clear lakes (Fig. 5D), where mean lake temperatures sometimes exceeded 15°C, *R_E_* sometimes exceeded *R_0_*. The same was true for simulations in mid-sized lakes when *R_0_* = 0.001 d^−1^. However, in all simulations, most of the *R_E_* values were below the *R_0_* values. The apparent hysteresis in *R_E_* for any one simulation results primarily from the change in temperature through the simulation.

**Figure 6:**
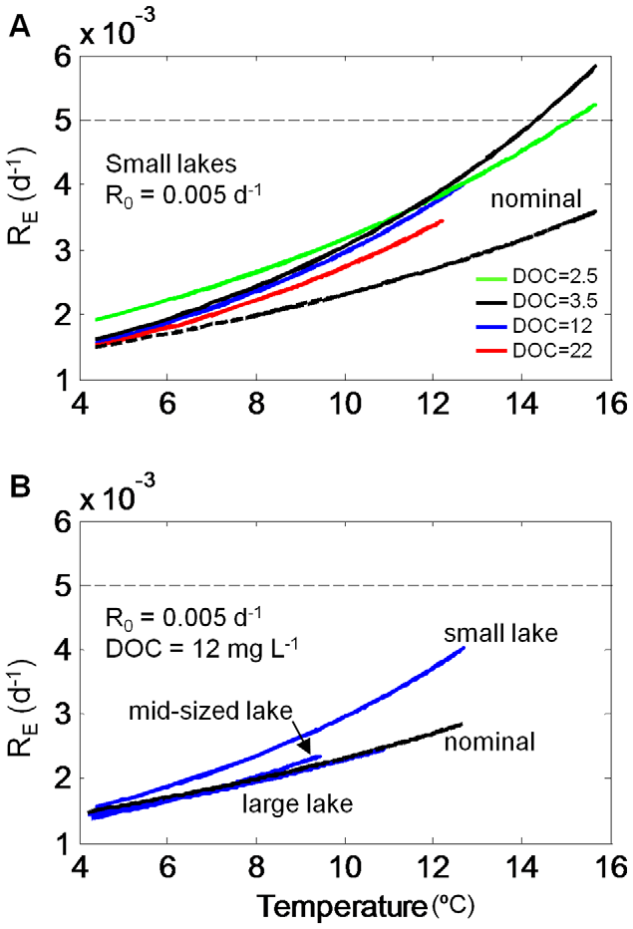
Effects of DOC and lake size on *R_E_*. Solid lines are exponential curves fit to data in Figure 5D–F for visual clarity. Dashed lines are *R_0_* (0.005 d^−1^) scaled by temperature according to the Arrhenius scaling function in the model. (A) For small lakes, water clarity elevates *R_E_* above values scaled by temperature alone. Differing DOC controls *R_E_* primarily through temperature, but clarity as well. (B) For three different lake sizes and DOC of 12 mg L^−1^, curves show that smaller lakes have elevated *R_E_*.

*R_E_* increased exponentially with temperature, and is fit well with a classical Arrhenius equation. We fit the Arrhenius equation to data in Figure 5D–F solely for the purpose of simplifying the data for each simulation. In Figure 6, we display a subset of those data to illustrate key points. In 6A, we plot scenarios of small lake area, constant *R_0_* = 0.005 d^−1^, and the full range of DOC levels, represented by the four different lines. All simulations produced *R_E_* above what would have been expected by temperature alone (dashed line). Only in the two low-DOC simulations did temperature exceed 15°C, and in those simulations *R_E_* met or exceeded *R_0_* at higher temperatures. DOC had a profound effect on water temperature, presumably through changing water clarity. It also appears that differences among simulations altered scaling of *R_E_*. In Figure 6B, *R* *_0_* and DOC were held constant at the mid-level values, and lake area was varied. Lake area influenced lake temperature, resulting in the highest *R_E_* in small lakes. The lake area effect on *R_E_* scaling was similar in magnitude to the DOC concentration effect seen in Figure 6A.

**Figure 7:**
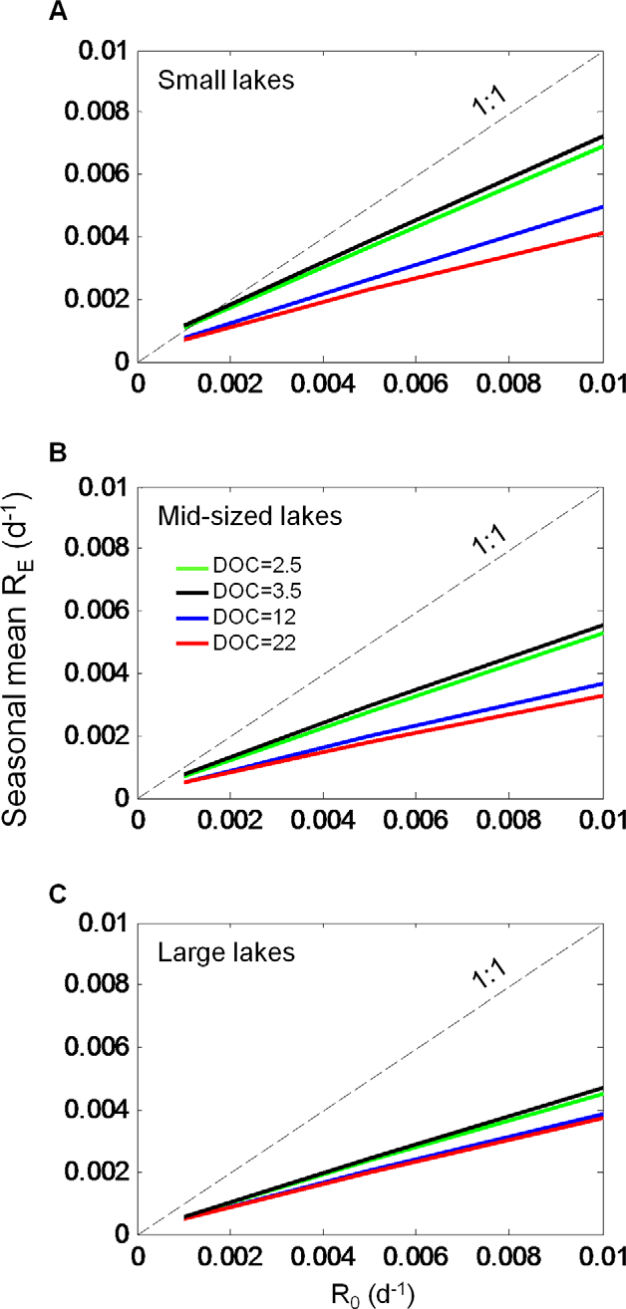
Seasonal mean *R_E_* versus *R_0_*.

At the seasonal scale, mean *R_E_* was consistently lower than *R_0_*. When seasonal mean *R_E_* was plotted against *R_0_*, the slope of the lines was less than 1 (Fig. 7), indicating that as *R_0_* increases, *R_E_* increases more slowly. If it were just the recalcitrance of the load that determined its fate, we would see *R_E_* equal *R_0_*, and *R_E_* would fall on or parallel to the 1∶1 line, but it does not. *R_E_* increases at a rate less than that of *R_0_* and near linearly across an order of magnitude in recalcitrance (0.001–0.01 d^−1^). The lines do not pass through the origin, however, because *R_E_* also includes photo oxidation of DOC, which in this model we do not covary with *R_0_*. Seasonal mean *R_E_* exceeded *R_0_* only in the small lakes when *R_0_* was at its lowest in the low DOC simulations. The DOC concentration effect was greatest in the small lakes under low-DOC simulations (Fig. 7A), where higher temperatures (Fig. 6A) led to a corresponding increase in *R_E_*.

**Figure 8:**
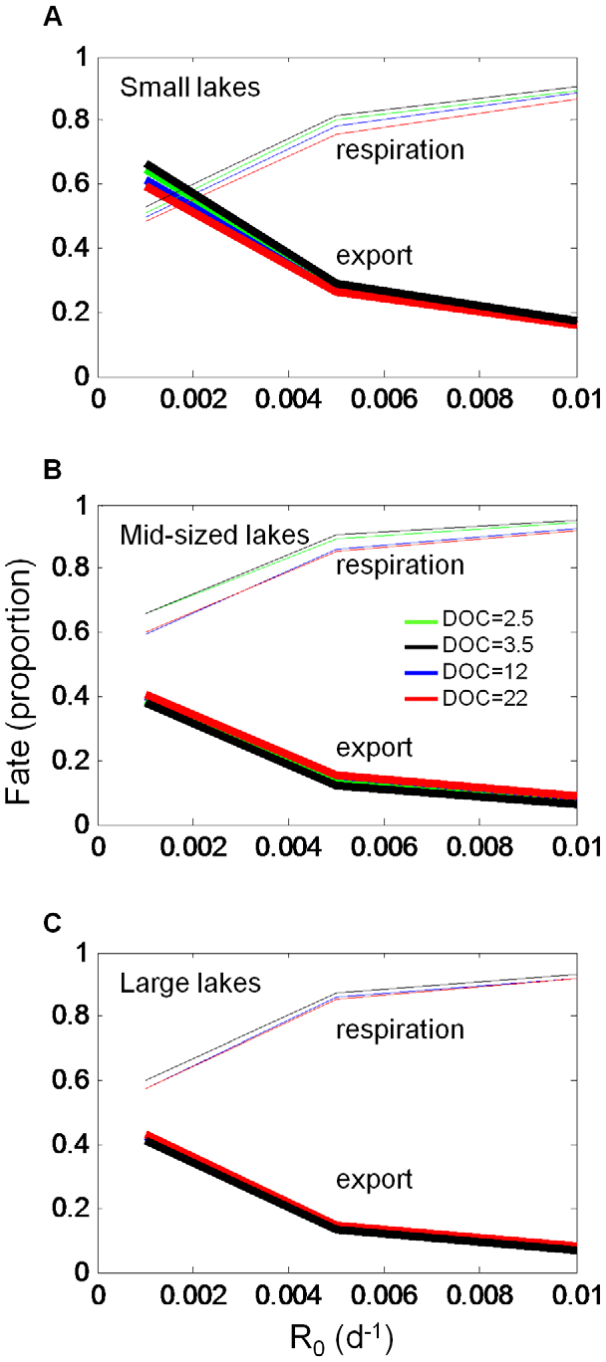
Fate of DOC as a function of *R_0_*.

The fate of the organic carbon in lakes was partitioned primarily between respiration and export, and was influenced by *R_0_*. Lake area and the corresponding hydrologic residence time had a strong influence on the fate of OC as being exported or respired (Fig. 8). In the midsized and larger lakes (Fig. 8B,C), most OC was respired. At *R_0_* values of about 0.005 d^−1^ and higher, more than 80% of the OC load was respired. Only in the smaller lakes at very low *R_0_* (Fig. 8A) did export exceed respiration. DOC concentration had a minor influence on the fate of OC, even though it was important in determining *R_E_* (e.g., Fig. 6A).

**Figure 9:**
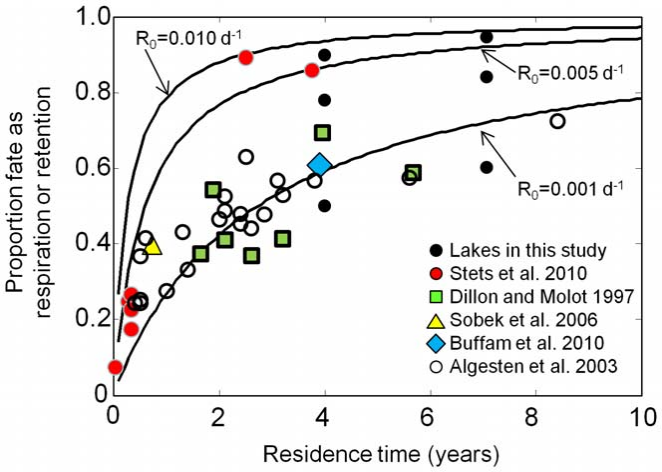
Proportion of fate as *R* (or retention) versus water residence time. Lines are calculated under the assumption that *R_0_* represents true ecosystem respiration and that *R_0_* and export, as calculated from residence time, are the only two fates of DOC. The two groups of black dots correspond to fates from the small lakes and large lakes simulations, when *R_0_* is set to the three respiration rates used to generate the lines. The colored markers are from studies that have quantified loads and export or respiration, as well as hydrologic residence time in northern latitude lakes. Values from the literature represent retention (retention = mineralization+sedimentation).

In our study, the balance between three factors – residence time, the choice of *R_0_*, and lake characteristics, primarily temperature and DOC concentration – controlled the fate of allochthonous DOC. For illustrative purposes, let us assume that export (*E*) and mineralization (*R*) are the dominant fates of OC loads to lakes. We can simply calculate the fate of OC as *R* for lakes of different residence times for a number of *R_0_* values (Fig. 9, solid lines). The large lakes in this study have a residence time of ca.7 years, which approximates to daily export rate of 0.0004 d^−1^. When we choose an *R_0_* of 0.005 d^−1^, then the fate of DOC would be 93% as *R* and 7% as *E* (i.,e., proportion *R* = 0.005/(0.005+0.0004) = 0.93). However, in our simulations and under the above conditions the proportion of fate as *R* is closer to 84% (Fig. 8C, and center black dot of the right group on Fig. 9). In effect, lake characteristics other than residence time have lowered the fate as *R* by about 9% by reducing seasonal mean *R_0_* from 0.005 d^−1^ to a seasonal *R_E_* of 0.002 d^−1^ (Fig. 7C). If we repeat the above thought experiment, but with the small lakes that have a residence time of ca. 4 years, then expected fate as *R* would be 88%, based on *R_0_* of 0.005 d^−1^. As we see in Figure 8A and the center black dot in the left group of dots in Figure 9, fate as *R* in our simulations is closer to 78%, and again, the reduction by 10% is due to *R_0_* being reduced to an *R_E_* of about 0.0025 d^−1^ (Fig. 7B).

## Discussion

Substantial research in the past decade has characterized lakes as hot spots of carbon cycling in the landscape, acting both as conduits of inorganic carbon and mineralization sites for terrigeneous organic carbon [2], [10], [12]. As the environment is subject to increasing levels of land use change and climate change, we might expect an increase in the frequency of extreme disturbances at the landscape scale [17]. Disturbances may be manifested, in part, by increased fluxes of nutrients through watersheds. How might lakes process these nutrients, especially organic carbon? The results of this study highlight the importance of understanding both the nature of the load, in terms of the recalcitrance of the organic carbon, and the physical characteristics of the lake. Here, we focus discussion on the interplay between those two important components and the implications for carbon cycling.

### Importance of the lake physical characteristics

Differences in size determine the extent to which lakes process OC loads. The most obvious consequence of lake size in this model is its effect on hydrologic residence time. Larger lakes in this region tend to have longer residence times, and the longer that DOC is resident in a lake, the more opportunity there is for that OC to be mineralized (Fig. 8C) [18]. Drawing inferences about OC processing rates from observational data remains challenging at the ecosystem scale due to the difficulties in measuring key hydrologic and DOC fluxes. However, a landmark study by *Dillon and Molot* [16] provides a basis for comparison. Although hydrologic residence times are longer in our lakes than in theirs, extrapolating the relationship between export and load from their study suggests that our mid-sized lakes should export about 15% of their loads, and indeed this estimate is reasonably close for values of *R_0_* near 0.005 d^−1^ (Fig. 8B). For the smaller lakes in our study, we do not have well constrained estimates of hydrologic residence times, but if we estimate them to be equal to the mean depth in years (a rough approximation for groundwater fed lakes in this region), we would expect about 40% export, based on *Dillon and Molot* [16]. Our estimates of export for smaller lakes range from about 20–40%, suggesting that other factors, such as higher DOC concentrations, play a role in the OC processing.

Lake DOC concentration appears to influence DOC processing. Most lakes in this region are strongly stratified for much of the open water season, cover broad ranges in temperature and nutrients, and have chromophoric compounds that attenuate irradiance [19]. More highly stained lakes (i.e., those with higher concentrations of recalcitrant DOC), such as TB, tend to be cooler [20], with obvious effects on the kinetics of mineralization. Darker lakes are also more strongly stratified with shallower mixed layer depths [21] and with cooler more anoxic hypolimnia [19]. Indeed, in this study the mean temperature of the smallest low-DOC lake (about 18°C) was nearly double that of the smallest high-DOC lake. Lakes with high DOC concentrations have low light penetration, which restricts depth of mineralization of OC [22] directly through photooxidation and indirectly through increasing the lability of recalcitrant OC [15]. We did not adjust photolytic decay parameters with load recalcitrance. However, in real ecosystems, it may be reasonable to expect these two rates to covary [15]. The importance of photolytic decay is difficult to gauge at the ecosystem scale. Although DOC degradation occurs near the surface of the lake, resulting in photo-oxidation of DOC or conversion of DOC to a much more labile state [15], rapid attenuation of light, especially ultraviolet radiation, decreases photolytic decay rates deeper in the water column [22]. At an ecosystem scale, photo-oxidation has been found to account for about 10% of total mineralization [23]. Clearly, the interactions between photo-oxidation, photolytic decay to a more labile state, and bacterial respiration warrant more careful study.

### Importance of the recalcitrance of the load

The recalcitrance of the load interacts with the lake physical characteristics to determine load fate. Recalcitrance is represented by a single parameter in this study, *R_0_*. What does the recalcitrance number, *R_0_*, really mean? Rates from past work span roughly an order of magnitude, suggesting uncertainty from a variety of factors, including the source of the OC and the method for estimating the rates [24]. For example, in a mass balance study of a Swedish lake, daily mineralization of OC was found to be about 0.001–0.003 d^−1^ [7]. In a study of lake water DOC from a north temperate system, *Houser* [25] found the mean degradation rate to be about 0.005 d^−1^. In laboratory incubations, dark bottle decay has been found to be about 0.0035 d^−1^ [15] or as high as 0.016 d^−1^ [26], whereas long-term degradation experiments have estimated decay rates to be closer to 0.0007 d^−1^ for river DOC [27] or 0.0008 d^−1^ for DOC derived from a wetland [28]. For the ranges given above (∼0.0007–0.016 d^−1^), there would be an approximate five-fold change in *R_E_* at the whole-lake scale (Fig. 7).

The value chosen for *R_0_* has substantial bearing on the estimated fate of DOC at the ecosystem scale. For lakes with a residence time of two years, the fate as *R* could be as low as 40% or as high as 85% (Fig. 9), depending on the value chosen for *R_0_*. However, results from other studies that focused on carbon loads and exports, rather than respiration rates, may help us constrain *R_E_*. In Figure 9, we plot the proportion of DOC fate as *R* from this study and from five other studies in which lakes are from a similar latitude, hydrologic residence time has been estimated, and *R_0_* was not simply assumed but measured as part of the study or was inferred by us from loads and exports. For literature values, we plot carbon retention (retention = mineralization+sedimentation) because the balance between mineralization and sedimentation is not always well quantified (Fig. 9). Dillon and Molot [16] calculated retention of DOC as the difference between stream inputs and outputs from their lakes. Estimates from their study benefit from well-defined inflows and outflows, but for many seepage lakes, such as those typical of northern Wisconsin, inputs are diffuse and difficult to measure. In Stets et al. [26], *R_0_* was determined from laboratory experiments and scaled to the ecosystem using an Arrhenius temperature function. They assumed sedimentation was negligible. Sobek et al. [7] provide a comprehensive organic carbon budget for their lake, including contributions to DOC by emergent macrophytes. We cannot separate respiration of macrophyte DOC from that of other OC sources, so as a first order approximation we assume the same rate and plot the proportion fate as *R*, which equates to 0.4. Buffam et al. [29] estimated carbon loads to lakes at the regional scale, and the plotted value represents their median carbon retention. Finally, Algesten et al. [18] estimated both the organic carbon loads and exports from lakes. Of the studies plotted in Fig. 9, Stets et al. [26] clearly has the highest fate as respiration. Their estimate for *R_0_* from bottle incubations was 0.016 d^−1^, which is high relative to other studies. The values of fate of DOC as *R* from their study fall below our *R_0_* = 0.010 d^−1^ line, probably because they adjusted their *R_0_* for temperature using a scaling function similar to ours. What we find striking about Figure 9 is that most literature values fall near the *R_0_* = 0.001 d^−1^ line. Because the plotted data are retention, which includes sedimentation, they likely overestimate *R*. If we were to adjust retention in these studies by removing the sedimentation component, which may range from 13–44% [9], [18], [29] or even as high as 50% in small humic lakes [11], then the literature data would tend to fall just below the *R_0_* = 0.001 d^−1^ line, which corresponds reasonably well to results from lakes in this study, represented by the lowest black dots in Fig. 9. The shape and magnitude of the lowest curve in Fig. 9 is remarkably similar to that determined by Curtis [30] in an empirical analysis of dissolved organic matter retention for Ontario lakes.

Uncertainty in *R* can lead to uncertainty in carbon load estimates for lakes. For most lakes, we do not have well-constrained estimates of the OC loads or their mineralization rates. If, for example, we assume fixed values for *R* and *E* in Equation 1, rearrange to solve for the loads (*I*), then we can calculate the DOC load necessary to produce the observed lake DOC concentrations through time, according to:
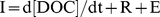
(3)Under steady state conditions, uncertainty in *R* and *E* manifests directly in uncertainty in *I*. As we see in Figure 9, a small change in *R_0_* or residence time, especially for lakes with residence times <c. 3 years, has a big impact on the fate of DOC. For lakes in our study, lowering *R_0_* from 0.005 d^−1^, the value used in calibration, to 0.001 d^−1^ requires a reduction in the loads of 30–60%, depending on the lake, to approximate the steady state used in calibration. Thus, the selection of an ecosystem-scale mineralization rate of DOC, while seemingly innocuous, has a large bearing on the magnitude of DOC load required to balance the lake OC budget.

For lakes in the Northern Highland Lake District of Wisconsin, we also have much to learn about the timing and magnitude of OC loads, which probably exert the highest uncertainty in lake carbon budgets [7]. In temperate zones, autumnal leaf litter fall tends to dominate the particulate C load from terrestrial systems to lakes [31]–[33]. However, a continuous input of fine particulates during summer [34] and pollen in spring [35], [36] supplements the more episodic, autumnal inputs. While leaf litter is a major source of terrestrial OC to lakes, forest canopies also entrain atmospheric deposition that can then be deposited during autumnal litterfall, or as throughfall, during episodic rain events [37]. The medley of litter types and atmospheric carbonaceous compounds may account for variability in timing, magnitude, and quality among these various sources of terrestrial carbon. Furthermore, variability in surface flows that deliver allochthonous DOC, as well as changing water levels in lakes, would affect the dynamics of lake carbon budgets. For most lakes in the NHLD, which on average have a hydrologic residence time of about four years [3], we doubt that subannual loading dynamics would impart much observable pattern on lake DOC. However, for small lakes with very short hydrologic residence times, a DOC pulse commensurate with a hydrologic pulse may be mostly exported if the magnitude of the hydrologic pulse approaches the lake volume. Such episodic events are not captured in this study but are worthy of further exploration and can be modeled using our current approach, provided there are adequate inflow measurements.

Our simplification of the carbon budget by focusing on allochthonous DOC leaves additional issues to be addressed. A full accounting of a lake's carbon budget would need to include the aforementioned fluxes of particulate organic carbon, sedimentation of autochthonous primary production, especially in highly eutrophic systems [38], and even flocculation of DOC in boreal lakes [11]. Do these fluxes alter the fate of allochthonous DOC? Certainly flocculation would, although the magnitude of this flux is not well quantified for a broad range of lakes. For example, Wachenfeldt and Tranvik [11] found sedimentation flux of DOC in highly humic lakes to be about 0.02 d^−1^, which greatly exceeds the upper end of *R_0_* tested in this study. However, it is not clear the extent to which this carbon is permanently buried in the sediments. If DOC sedimentation and permanent burial is a flux much greater than respiration, it would either require an additional load of allochthonous DOC to balance the overall budget or remove most of the observable DOC from the water column. High-DOC lakes in this study are at the upper end of the range found in Wisconsin, where most of the water volume at a regional scale is in larger lakes with lower DOC concentrations [19]. For lakes with relatively low DOC concentrations, organic matter buried in sediments is thought to derive primarily from particulate autochthonous sources, such as phytoplankton and macrophytes [39]. An additional challenge in larger lakes may be separating in our observed data the allochthonous and autochthonous contributions to DOC pools and fluxes, because values are relatively low.

Recent understanding of the importance of freshwater systems in continental-scale carbon cycling compels us to better understand underlying mechanisms of DOC processing in lakes [9]. Although empiricism has helped explain relationships between lake DOC and geologic setting, land cover, and local climate [40], [7], well-constrained quantification of the magnitude and quality of loads to lakes remains elusive. Predicting the fate of DOC in lakes under changing climate and land use requires better quantification of DOC mineralization and export rates. DOC respiration has been inferred indirectly from measures of CO_2_ concentration [41] or scaled -up from bottle experiments assumed to be applicable at the ecosystem scale [10], [22], [23], [42]. However, we are unaware of any study that has explored the controls on DOC mineralization in a range of lake types. A unique feature of the present study is the use of broad gradients of key lake features to better understand the relative importance of DOC quality and lake characteristics in determining the fate of DOC in lakes.

**Figure 10:**
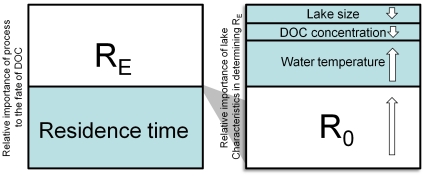
Factors controlling the fate of DOC loads to lakes. (Left) For a broad range of lakes in the NHLD, both residence time and ecosystem respiration (*R_E_*) can have near equal influence over the fate of DOC loads to lakes. (Right) The controls of *R_E_* depend primarily on assumed recalcitrance (*R_0_*) and water temperature, however, DOC concentration and lake size exert minor control as well. The overall effect on *R_0_* for lakes in this study was to reduce it by approximately 50%. The effects on fate as respiration are less dramatic because of the importance of residence time in the governing equation.

We have seen emerge from this complex suite of physical, chemical, and biological processes a relatively small number of factors that exert primary control over the fate of allochthonous DOC in lakes (Fig. 10). Hydrologic residence time and *R_E_* appear to be equally important to the fate of DOC in lakes with residence times of roughly 2–4 years, assuming a relatively high level of recalcitrance in DOC (Fig. 9, *R_0_* = 0.001 d^−1^). Naturally, higher rates of *R_0_* tip the balance of fate more toward respiration; however, results from other studies suggest *R_0_* may be closer to the bottom end of the range. Temperature is important in scaling *R_0_* to *R_E_*, and may lower *R_0_* dramatically (∼50%, Fig. 7). However, in determining the fate of DOC, effects of temperature are dampened by the overall importance of residence time, and to a lesser extent the effects of lake size and DOC on mixing and photo-oxidation. Encoding these factors – residence time, *R_0_* and water temperature, estimated from lake area and DOC concentration – in simplified models parameterized over a larger gradient of lake types would be a substantial advancement toward modeling lake carbon cycling to obtain whole-lake carbon budgets that are necessary to better understand the contributions of lakes to carbon cycling at the landscape scale.

## Materials and Methods

We use a coupled hydrodynamic–biogeochemical model calibrated to observed data from four lakes in northern Wisconsin to study the fate of DOC loads to lakes. Data are from 2006, and we focus on the open water period that spans roughly April through October. Our first goal is to study different DOC recalcitrance values on the fate of DOC loads, but not the magnitude of the loads themselves. Therefore, we fix the magnitude of carbon loads to the lakes and vary the assumed recalcitrance level of DOC, represented here by a first-order decay rate (*R_0_*). Our second goal is to determine how lake characteristics, such as temperature, water clarity, and wind-driven mixing, alter *R_0_*. Finally, we assess the fate of the DOC load as being mineralized or exported downstream. We use four lakes that cover broad ranges in lake area and DOC concentration (Fig. 1) as calibration systems within an ensemble of 48 simulations. Finally, we compare our estimates of the fate of DOC loads to lakes with those from the literature.

### Study lakes

The four calibration lakes in this study are primary study lakes of the North Temperate Lakes Long Term Ecological Research program (NTL-LTER) [42]. These lakes are located in the Northern Highland Lake District of Wisconsin, and are characterized by moderate to low acid neutralizing capacity (ANC), conductivity, and productivity. The lakes were chosen for their contrasts in morphometry and concentrations of carbon and phosphorus (Table 1). These variables are known to affect mixed layer depth [21], and ecosystem primary productivity and respiration [43]. Organic carbon-rich lakes may be especially responsive to photobleaching [22] and photoxidation [13], particularly when the DOC originates from terrestrial sources [15]. These characteristics likely influence how lakes process OC pulses and how they respond to meteorological forcing. Crystal Bog Lake (CB) and Trout Bog Lake (TB) are small dystrophic lakes with moderate to high dissolved organic carbon (DOC) and total phosphorus (TP) concentrations. Sparkling Lake (SP) and Trout Lake (TR) are large and deep oligotrophic lakes with low DOC and TP concentrations. Physical and chemical data pertaining to these lakes were collected by the NTL LTER program in 2006. Analytical methods for these variables are described at http://lter.limnology.wisc.edu.

**Table 1:** Limnological characteristics for the four calibration lakes.

Lake	Area (ha)	Mean depth (m)	Hydrologic Residence Time (yr)*	T (°C)	DOC (mg L^−1^)	DIC (mg L^−1^)	TP (µg L^−1^)
Crystal Bog Lake (CB)	0.5	1.7	1.7	10.7	9.3	2.0	19.8
Trout Bog Lake (TB)	1.1	5.6	5.6	10.1	21.0	3.8	38.9
Sparkling Lake (SP)	64.0	10.9	10	10.7	3.3	8.8	15.1
Trout Lake (TR)	1607.9	14.6	5	9.9	2.8	11.3	11.1

DOC, TP, and ANC are 2006 annual means integrated through the water column during the simulation period. T is hypsometrically weighted mean annual water temperature.

*Hydrologic residence time (yr) for SP and TR from Ackerman [48], and for CB and TB were assumed to equal mean depth (m).

### The model

To simulate the lakes, we used DYRESM-CAEDYM (DC; http://www.cwr.uwa.edu.au/services/models/legacy/model/dyresmcaedym/). DC couples one-dimensional hydrodynamics with a broad collection of chemical and biological processes to simulate mixing, transport, and transformational processes at high vertical (<1 m) and temporal (<1 d) resolution [44]–[46]. DC has been used in a wide range of water quality and ecosystem applications, including an ecosystem study in our region [47]. We chose a one-dimensional model because the study lakes strongly stratify during summer, leading to marked vertical gradients in the characteristics important to DOC synthesis and degradation, such as light, nutrient concentrations, and temperature. Further, our observational data are one-dimensional, based on profiles of a central monitoring station, and the primary dynamic of interest was seasonal rather than spatial change. DC was not designed to model ice-covered lakes, so we limited our analyses to the open-water season.

Meteorological and inflow data drive the model, beginning with starting conditions in April based on observed limnological profiles. Wind speed was measured by sensors mounted on a buoy located in the deepest part of the lake. Irradiance, both short- and long-wave, as well as precipitation, were measured at a nearby (<5 km) weather station. Hydrologic residence times were from previous studies or assumed to equal mean depth for the bog lakes, CB and TB (Table 1). For lakes with no well-defined defined surface flow, hydrologic residence time is closely related to mean depth because annual inflow due to precipitation and groundwater is about 1 m [48]. Lakes in this study have low productivity [42], therefore, nutrient loads (nitrogen and phosphorus) were set to low values. All inflows and loads were held constant through the simulation. Although this simplifying assumption under-represents natural time variability, it allows us to attribute DOC dynamics to meteorology and internal lake processes. We approximated DOC loads from previous modeling results [10], adjusting them slightly to ensure near steady-state of DOC concentration in each calibration lake through the simulation. Model output is displayed at daily time-steps and aggregated to mean epilimnetic and mean hypolimnetic values to allow for easy comparison with observational data. A number of parameters control physical and chemical (Table S1), phytoplankton (Table S2), and zooplankton (Table S3) processes in DC. In our study, nearly all parameters were set to values from the literature and were assumed to be the same for all lakes. To greatly simplify the phytoplankton dynamics of the lakes, we modeled four phytoplankton functional groups, assuming mid-range parameter values for growth, death, and sedimentation. The model was calibrated to DOC and temperature observational data by adjusting the aforementioned parameters until predictions best matched observations. Visual inspection of predictions and data was used to assess goodness of fit. Data were not weighted by volume, unless otherwise specified. We fit to mixing depth and timing of mixing to provide an indication of the fit of the model for temperature and to verify mixing dynamics were adequately captured, in common with previous use of DC [47]. We also provide Spearman's rank correlation coefficient as a quantitative metric of fit.

Many of the results in this study are described as “fates” of the organic carbon input. Fate is defined as the sum total over the simulation of DOC that has been mineralized or respired (R, all forms of mineralization) or exported from the lake via water flow (E). Because external DOC loads were adjusted to produce near steady-state DOC in the lakes, we do not consider changes in standing stock as a fate. Rather, we subtract any small changes in standing stock from the loads and use that result as the total load, which always equals the sum of respiration and export.

One focus of this study is to compare assumed rates of respiration, with the actual total ecosystem respiration. The assumed rate is represented by a first order decay parameter, typically measured in laboratory experiments and standardized to 20°C, and we label that rate *R_0_* (d^−1^). During simulations, *R_0_* is scaled in the model according to the general biogeochemical temperature scaling function:
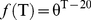
(2)where *T* is the observed temperature in °C and *θ* is 1.073, which equates to a Q10 value of 2.0 when scaled according to the exponent *T* with reference to 20°C. Because the model tracks vertical temperature gradients, *R_0_* scaled to the ecosystem scale will reflect the vertical temperature of the lake. The model also tracks photo-oxidative losses, which can be added to mineralization losses due to R. Thus, the effective daily respiration at the ecosystem scale is the sum of all forms of DOC mineralization. In this study, we calculate daily DOC mineralization simply through changes in mass balance and term that ecosystem respiration (*R_E_*; d^−1^). Our goal is not to study the form or parameterization of equation 2, but rather how our choice of *R_0_*, in balance with lake characteristics, results in *R_E_*. Furthermore, we are interested in how variations in *R_E_* influence the fate of the DOC load as being respired or exported from the ecosystem.

### Scenarios

To test the effects of lake size, lake DOC state, and assumed value of respiration (*R_0_*) on *R_E_* and the fate of DOC loads, we create simulations orthogonal in those characteristics. Figure 1 shows the 16 simulations that cover broad ranges in lake area and DOC concentration. The shaded boxes indicate the actual areas and mean DOC concentrations for the four calibration lakes. For each of the 16 simulations, we tested three values of *R_0_*: 0.001, 0.005, and 0.010 d^−1^. Thus, the total number of simulations was 48. These values of *R_0_* are reasonably representative of the range documented in the literature for laboratory DOC degradation experiments. We give a more complete description of the literature values of *R_0_* in the Discussion.

## Supporting Information

Table S1 **General model parameters.**
(DOC)

Table S2 **Phytoplankton parameters.**
(DOC)

Table S3 **Zooplankton parameters.**
(DOC)
