# Supplementary material for: Fate of Allochthonous Dissolved Organic Carbon in Lakes: A Quantitative Approach
Source: PLoS One. 2011 Jul 14;6(7):e21884. doi: 10.1371/journal.pone.0021884 (PMC3136486; doi:10.1371/journal.pone.0021884)
Supplement: Table S1 — General model parameters. (DOC) [file pone.0021884.s001.doc]

**Table S1.**

| **Parameter** | **Description** | **Units** | **Value** |
| --- | --- | --- | --- |
|  | Light extinction coefficient of pure water | m^-1^ | 0.25 |
|  | Fraction of incoming solar radiation which is photosynthetically active | - | 0.45 |
|  | Specific light attenuation coefficient due to the action of labile DOC | m^-1^ (gC m^-3^)^-1^ | 0.02[[1]](#endnote-2) |
|  | Specific light attenuation coefficient due to the action of refractory DOC | m^-1^ (gC m^-3^)^-1^ | 0.001[[2]](#endnote-3) |
|  | Specific light attenuation coefficient due to the action of labile POC | m^-1^ (gC m^-3^)^-1^ | 0.01Error: Reference source not found |
|  | Specific light attenuation coefficient due to the action of refractory POC | m^-1^ (gC m^-3^)^-1^ | 0.02 |
|  | Maximum sediment oxygen demand (SOD) at 20C | g m^-2^ day^-1^ | 0.46[[3]](#endnote-4),[[4]](#endnote-5) |
|  | Half saturation constant for DO effect on SOD | g DO m^-3^ | 1.5^Error: Reference source not found^ |
|  | Temperature multiplier for SOD | - | 1.08[[5]](#endnote-6) |
|  | Equivalent DO at the air-water interface | g DO m^-3^ | Error: Reference source not found |
|  | Oxygen transfer coefficient dependent on wind speed | m s^-1^ | Error: Reference source not found |
|  | Partial pressure of CO_2_ at the air-water interface | atm | 350e-6 |
|  | Gas transfer velocity for CO_2_ | m s^-1^ | Error: Reference source not found |
|  | Ion product of water |  | [[6]](#endnote-7) |
| **;** | First and second acidity constants |  | Error: Reference source not found |
|  | Stoichiometric ratio of DO to C during photosynthesis and respiration | g DO (g C)^-1^ | 2.67 |
|  | Stoichiometric ratio of DO to N during nitrification | g DO (g N)^-1^ | 3.43 |
|  | Settling velocity of particulate detritus (POM), used for POC, PON, POP | m s^-1^ | Calculated from Stoke’s Law: |
|  | Diameter of POM particles | m | 8.0e-05 |
|  | Density of POM particles | kg m^-3^ | 1040[[7]](#endnote-8) |
|  | Maximum rate of POC decomposition to DOC at 20C | day^-1^ | 0.070[[8]](#endnote-9) |
|  | Maximum rate of POP decomposition to DOP at 20C | day^-1^ | 0.030[[9]](#endnote-10) |
|  | Maximum rate of PON decomposition to DON at 20C | day^-1^ | 0.035Error: Reference source not found |
|  | Maximum denitrification rate under anoxia at 20C | day^-1^ | 0.05Error: Reference source not found |
|  | Temperature multiplier for denitrification | - | 1.05Error: Reference source not found |
|  | Half saturation constant for denitrification dependence on oxygen | g DO m^-3^ | 0.4Error: Reference source not found |
|  | Maximum nitrification rate under oxygen saturation at 20C | day^-1^ | 0.106Error: Reference source not found^,Error: Reference source not found^ |
|  | Temperature multiplier for nitrification | - | 1.08Error: Reference source not found |
|  | Half saturation constant for nitrification dependence on oxygen | g DO m^-3^ | 1.5Error: Reference source not found |
|  | Temperature multiplier for sediment nutrient fluxes | - | 1.08 |
|  | Maximum release rate of PO_4_ from sediments at 20C | g m^-2^ day^-1^ | 0.0125Error: Reference source not found,[[10]](#endnote-11),[[11]](#endnote-12) |
|  | Half saturation constant for sediment PO_4_ release dependence on DO | g DO m^-3^ | 2.0Error: Reference source not found |
|  | Maximum release rate of NH_4_ from sediments at 20C | g m^-2^ day^-1^ | 0.31Error: Reference source not found |
|  | Half saturation constant for sediment NH_4_ release dependence on DO | g DO m^-3^ | 2.0Error: Reference source not found^,Error: Reference source not found^ |
|  | Maximum release rate of NO_3_ from sediments at 20C | g m^-2^ day^-1^ | -0.12Error: Reference source not found |
|  | Half saturation constant for sediment NH_4_ release dependence on DO | g DO m^-3^ | 50Error: Reference source not found |
|  | Maximum release rate of DOC from sediments at 20C | g m^-2^ day^-1^ | 0.0Error: Reference source not found |
|  | Maximum release rate of DOP from sediments at 20C | g m^-2^ day^-1^ | 0.0Error: Reference source not found |
|  | Maximum release rate of DON from sediments at 20C | g m^-2^ day^-1^ | 0.0Error: Reference source not found |
|  | Half sat constant for sediment DOC release dependence on DO | g DO m^-3^ | 0.5^Error: Reference source not found^ |

1. Kirk JTO (1994) Estimation of the absorption and the scattering coefficients of natural waters by use of underwater irradiance measurements. Applied Optics 33: 3276-3278. [↑](#endnote-ref-2)
2. Morris DP, Zagarese H, Williamson CE, Balseiro EG, Hargreaves BR, et al. (1995) The attentuation of solar UV radiation in lakes and the role of dissolved organic carbon. Limnology and Oceanography 40: 1381-1391. [↑](#endnote-ref-3)
3. Brock TD (1985) A Eutrophic Lake, Lake Mendota, WI; Billings WD, Lange OL, Remmert H, editors. New York: Springer-Verlag. 308 p. [↑](#endnote-ref-4)
4. Derived from NTL LTER data. [↑](#endnote-ref-5)
5. Wanninkhof R (1992) Relationship between wind-spped and gas-exchange over the ocean. Journal of Geophysical Research-Oceans 97: 7373-7382. [↑](#endnote-ref-6)
6. Butler JN (1982) Carbon dioxide equilibria and their applications: Addison-Wesley. x, 259 p. p. [↑](#endnote-ref-7)
7. Degobbis D, Gilmartin M (1990) Nitrogen, phosphorus, and biogenic silicon budgets for the northern Adriatic Sea. Oceanologica Acta 13: 31-45. [↑](#endnote-ref-8)
8. Sinsabaugh RL, Findlay S (1995) Microbial production, enzyme activity, and carbon turnover in surface sediments of the Hudson River estuary. Microbial Ecology 30: 127-141. [↑](#endnote-ref-9)
9. Jorgensen SE, Bendoricchio G (2001) Fundamentals of Ecological Modelling. Oxford, UK: Elsevier Science [↑](#endnote-ref-10)
10. Holdren GC, Armstrong DE (1980) Factors affecting phosphorus release from intact lake sediment cores Environmental Science & Technology 14: 79-87. [↑](#endnote-ref-11)
11. Serruya C, Edelstein M, Pollingher U, Serruya S (1974) Lake Kinneret sediments: nutrient composition of the pore water and mud water exchanges. Limnology and Oceanography 19: 489-508. [↑](#endnote-ref-12)
