# Supplementary material for: Fate of Allochthonous Dissolved Organic Carbon in Lakes: A Quantitative Approach
Source: PLoS One. 2011 Jul 14;6(7):e21884. doi: 10.1371/journal.pone.0021884 (PMC3136486; doi:10.1371/journal.pone.0021884)
Supplement: Table S2 — Phytoplankton parameters. (DOC) [file pone.0021884.s002.doc]

**Table S2.**

| Parameter | Description | Units | Microcystis | Aphanizomenon | Chlorophytes | Diatoms |
| --- | --- | --- | --- | --- | --- | --- |
|  | Maximum potential growth rate | day^-1^ | 0.6[[1]](#endnote-2) | 0.48[[2]](#endnote-3),[[3]](#endnote-4) | 0.2Error: Reference source not found,[[4]](#endnote-5),[[5]](#endnote-6) | 1.25[[6]](#endnote-7) |
| *I_s_* | Light saturation for maximum production | μEm^-2^s^-1^ | 250 | 220 | 170Error: Reference source not found | 20 |
|  | Specific attenuation coefficient | m^-1^ (gC m^-3^)^-1^ | 0.198 | 0.198 | 0.198 | 0.198[[7]](#endnote-8) |
|  | Half saturation constant for phosphorus uptake | g P m^-3^ | 0.0018 | 0.0012 | 0.01[[8]](#endnote-9),[[9]](#endnote-10) | 0.005Error: Reference source not found |
|  | Half saturation constant for nitrogen uptake | g N m^-3^ | 0.02 | 0.001 | 0.030 | 0.060 |
|  | Minimum internal N ratio | g N (g C)^-1^ | 0.070^Error: Reference source not found^ | 0.070Error: Reference source not found | 0.090Error: Reference source not found | 0.090Error: Reference source not found |
|  | Maximum internal N ratio | g N (g C)^-1^ | 0.24Error: Reference source not found | 0.16Error: Reference source not found | 0.14Error: Reference source not found | 0.15Error: Reference source not found |
|  | Maximum rate of nitrogen uptake | g N (g C)^-1^ day^-1^ | 0.08 | 0.12 | 0.060 | 0.15 |
|  | Minimum internal P ratio | g P (g C)^-1^ | 0.002Error: Reference source not found | 0.005Error: Reference source not found | 0.006Error: Reference source not found | 0.021Error: Reference source not found |
|  | Maximum internal P ratio | g P (g C)^-1^ | 0.023Error: Reference source not found | 0.023Error: Reference source not found | 0.059Error: Reference source not found | 0.085Error: Reference source not found |
|  | Maximum rate of phosphorus uptake | g P (g C)^-1^ day^-1^ | 0.01[[10]](#endnote-11) | 0.01 | 0.007Error: Reference source not found | 0.018Error: Reference source not found |
|  | N fixation rate | g N (g C)^-1^ day^-1^ | 0 | 0.15[[11]](#endnote-12) | 0 | 0 |
|  | Growth reduction under N fixation | - | 1.00 | 0.67 | 1.00 | 1.00 |
|  | Temperature multiplier for growth | - | 1.07[[12]](#endnote-13) | 1.10 | 1.08Error: Reference source not found | 1.08 |
|  | Standard temperature | °C | 19 | 24 | 20 | 19 |
|  | Optimum temperature | °C | 30Error: Reference source not found | 30Error: Reference source not found,[[13]](#endnote-14) | 21[[14]](#endnote-15),Error: Reference source not found,Error: Reference source not found | 17Error: Reference source not found |
|  | Maximum temperature | °C | 40Error: Reference source not found | 40Error: Reference source not found | 35Error: Reference source not found^,Error: Reference source not found^ | 22Error: Reference source not found |
|  | Metabolic loss rate coefficient | day^-1^ | 0.05[[15]](#endnote-16) | 0.05 | 0.05Error: Reference source not found | 0.05Error: Reference source not found^,Error: Reference source not found^ |
|  | Temperature multiplier for metabolic loss | - | 1.10 | 1.09 | 0.06 | 1.08 |
|  |  | - | 0.014 | 0.014 | 0.014 | 0.014 |
|  | Fraction of respiration relative to total metabolic loss | - | 0.8 | 0.8 | 0.8 | 0.5 |
|  | Fraction of metabolic loss rate that goes to DOM | - | 0.3 | 0.1 | 0.1 | 0.5 |
|  | Cell diameter | m | 1.0e-5 | 1.0e-7 | 1.0e-5 | 1.0e-5 |
|  | Settling velocity | m s^-1^ | 3.6 e -5 | -5.1 e-7 | 1.2 e -6 | -0.057Error: Reference source not found |
| *Y_ChlC_* | Chlorophyll:C ratio | - | 50 | 100 | 40 | 40 |
| *Y_CBiovol_* | Carbon:biovolume ratio (used for estimating algal biomass gC/m^3^) | pg C μm^-3^ | 0.127Error: Reference source not found | 0.127Error: Reference source not found | 0.198Error: Reference source not found | 0.199Error: Reference source not found |

1. Reynolds C (2006) The Ecology of Phytoplankton; Usher M, Suanders D, Peet R, Dobson A, editors. New York: Cambridge University Press. [↑](#endnote-ref-2)
2. Foy RH, Gibson CE, Smith RV (1976) The influence of daylength, light intensity, and temperature on the growth rates of planktonic blue-green algae. British Phycological Journal 11: 151-163. [↑](#endnote-ref-3)
3. Fogg GE (1949) Growth and heterocycst production i*n Anabaena cylindrica Lemm I*I. in relation to carbon and nitrogen metabolism Ann Botany London 13: 241-259. [↑](#endnote-ref-4)
4. Pollingher U, Berman T (1982) Relative contributions of net and nano-phytoplankton to primary production in Lake Kinneret (Israel) Archiv fur Hydrobiologie 96: 33-46. [↑](#endnote-ref-5)
5. Sandgren C (1988) Growth and Reproductive Strategies of Freshwater Phytoplankton; Sandgren C, editor: Cambridge University Press. v, 442 p. p. [↑](#endnote-ref-6)
6. Butterwick C, Heaney SI, Talling JF (2005) Diversity in the influence of temperature on the growth rates of freshwater algae, and its ecological relevance. Freshwater Biology 50: 291-300. [↑](#endnote-ref-7)
7. Derived from NTL LTER data. [↑](#endnote-ref-8)
8. Zohary T, unpublished data (after Gal et al., 2009). [↑](#endnote-ref-9)
9. Sandgren (1995) Chrysophyte Algae: Ecology, Phylogeny, and Development: Cambridge University Press. xiv, 399 p. p. [↑](#endnote-ref-10)
10. Healey FP, Hendzel LL (1979) Indicators of phosphorus and nitrogen deficiency in 5 algae in cultures. Journal of the Fisheries Research Board of Canada 36: 1364-1369. [↑](#endnote-ref-11)
11. Dugdale VA, Dugdale RC (1962) Nitrogen metabolism in lakes. II. role of nitrogen fixation in sanctuary Lake Pennsylvania. Limnology and Oceanography 7: 170-177. [↑](#endnote-ref-12)
12. Hipsey et al. in preparation. [↑](#endnote-ref-13)
13. Imai H, Chang KH, Kusaba M, Nakano S (2009) Temperature-dependent dominance o*f Microcyst*is (Cyanophyceae) species*: M. aerugino*sa an*d M. wesenberg*ii. Journal of Plankton Research 31: 171-178. [↑](#endnote-ref-14)
14. Ukeles R (1961) The Effect of Temperature on the Growth and Survival of Several Marine Algal Species. Biological Bulletin 120: 255-264. [↑](#endnote-ref-15)
15. Robson BJ, Hamilton DP (2004) Three-dimensional modelling of *a Microcyst*is bloom event in the Swan River estuary, Western Australia. Ecological Modelling 174: 203-222. [↑](#endnote-ref-16)
