# Supplementary material for: Fate of Allochthonous Dissolved Organic Carbon in Lakes: A Quantitative Approach
Source: PLoS One. 2011 Jul 14;6(7):e21884. doi: 10.1371/journal.pone.0021884 (PMC3136486; doi:10.1371/journal.pone.0021884)
Supplement: Table S3 — Zooplankton parameters. (DOC) [file pone.0021884.s003.doc]

**Table S3.**

| Parameter | Descriptioon | Units | Predatory | Macro-large | Macro-small | Micro |
| --- | --- | --- | --- | --- | --- | --- |
|  | Grazing rate | gC m^-3^  (g Z m^-3^)^-1^ day^-1^ | 1[[1]](#endnote-2) | 0.75[[2]](#endnote-3),[[3]](#endnote-4) | 0.3Error: Reference source not found | 0.5 |
|  | Grazing efficiency | - | 0.8 | 0.7 | 0.8 | 0.85 |
|  | Respiration rate coefficient | day^-1^ | 0.1Error: Reference source not found | 0.2Error: Reference source not found,[[4]](#endnote-5) | 0.075[[5]](#endnote-6) | 0.025 |
|  | Mortality rate coefficient | day^-1^ | 0.02 | 0.04 | 0.015 | 0.005 |
|  | Fecal pellet fraction of grazing | day^-1^ | 0.025 | 0.05 | 0.02 | 0.007 |
|  | Excretion fraction of grazing | day^-1^ | 0.1Error: Reference source not found | 0.1Error: Reference source not found | 0.1 | 0.1 |
|  | Fecal pellet fraction that sinks directly to sediments | - | 0.7 | 0.1 | 0.1 | 0.1 |
|  | Temperature multiplier for growth | - | 1.07Error: Reference source not found | 1.07Error: Reference source not found | 1.07 | 1.07 |
|  | Standard temperature | °C | 20Error: Reference source not found | 20Error: Reference source not found | 20Error: Reference source not found | 20[[6]](#endnote-7) |
|  | Optimum temperature | °C | 19Error: Reference source not found,[[7]](#endnote-8) | 20Error: Reference source not found^,Error: Reference source not found^ | 18Error: Reference source not found | 24Error: Reference source not found |
|  | Maximum temperature | °C | 35Error: Reference source not found | 35Error: Reference source not found | 35 | 35 |
|  | Respiration temperature dependence | - | 1.11Error: Reference source not found | 1.15Error: Reference source not found | 1.06 | 1.06 |
|  | Half saturation constant for grazing | g C m^-3^ | 2[[8]](#endnote-9) | 2[[9]](#endnote-10) | 2 | 2[[10]](#endnote-11) |
|  | Internal ratio of nitrogen to carbon. | g N (g C)^-1^ | 0.18[[11]](#endnote-12),Error: Reference source not found | 0.18[[12]](#endnote-13) | 0.16[[13]](#endnote-14),Error: Reference source not found | 0.15[[14]](#endnote-15) |
|  | Internal ratio of phosphorus to carbon | g P (g C)^-1^ | 0.0045Error: Reference source not found^,Error: Reference source not found^ | 0.0125Error: Reference source not found^,Error: Reference source not found^ | 0.006Error: Reference source not found^,Error: Reference source not found^ | 0.03Error: Reference source not found |
|  | Preference of zooplankton for predatory zooplankton | - | 0.15[[15]](#endnote-16) | 0Error: Reference source not found,[[16]](#endnote-17) | 0Error: Reference source not found | 0Error: Reference source not found |
|  | Preference of zooplankton for macro- large zooplankton | - | 0.15Error: Reference source not found^,Error: Reference source not found^ | 0Error: Reference source not found^,Error: Reference source not found^ | 0Error: Reference source not found | 0Error: Reference source not found |
|  | Preference of zooplankton for macro-small zooplankton | - | 0.15Error: Reference source not found^,Error: Reference source not found^ | 0Error: Reference source not found | 0Error: Reference source not found | 0Error: Reference source not found |
|  | Preference of zooplankton for micro zooplankton | - | 0.15Error: Reference source not found^,Error: Reference source not found^ | 0Error: Reference source not found^,Error: Reference source not found^ | 0Error: Reference source not found | 0Error: Reference source not found |
|  | Preference of zooplankton for POC | - | 0Error: Reference source not found | 0.1[[17]](#endnote-18) | 0.098[[18]](#endnote-19) | 0.05 |
|  | Preference of zooplankton for Bacteria | - | 0Error: Reference source not found | 0.6Error: Reference source not found | 0.06Error: Reference source not found | 0.06Error: Reference source not found |
| *P_A Mic_* | Preference of zooplankton for *Microcystis*-like phyto | - | 0Error: Reference source not found | 0.01[[19]](#endnote-20) | 0.001Error: Reference source not found | 0.05Error: Reference source not found |
| *P_A Aph_* | Preference of zooplankton for *Aphaniazomenon*-like phyto | - | 0Error: Reference source not found | 0.01[[20]](#endnote-21) | 0.001Error: Reference source not found | 0.05Error: Reference source not found |
| *P_A Chlor_* | Preference of zooplankton for Chloro/chryso-like phyto | - | 0Error: Reference source not found | 0.15Error: Reference source not found | 0.075Error: Reference source not found | 0.05Error: Reference source not found |
| *P_A Diat_* | Preference of zooplankton for diatom-like phyto | - | 0Error: Reference source not found | 0.18Error: Reference source not found | 0.075Error: Reference source not found | 0.05[[21]](#endnote-22) |
| *Min_res_* | Minimum phytoplankton biomass below which zooplankton will not graze | g C m^-3^ | 0.01 | 0.03 | 0.8 | 0.05 |

1. Kirk JTO (1994) Estimation of the absorption and the scattering coefficients of natural waters by use of underwater irradiance measurements. Applied Optics 33: 3276-3278.

2. Morris DP, Zagarese H, Williamson CE, Balseiro EG, Hargreaves BR, et al. (1995) The attentuation of solar UV radiation in lakes and the role of dissolved organic carbon. Limnology and Oceanography 40: 1381-1391.

3. Brock TD (1985) A Eutrophic Lake, Lake Mendota, WI; Billings WD, Lange OL, Remmert H, editors. New York: Springer-Verlag. 308 p.

4. Wanninkhof R (1992) Relationship between wind-spped and gas-exchange over the ocean. Journal of Geophysical Research-Oceans 97: 7373-7382.

5. Butler JN (1982) Carbon dioxide equilibria and their applications: Addison-Wesley. x, 259 p. p.

6. Degobbis D, Gilmartin M (1990) Nitrogen, phosphorus, and biogenic silicon budgets for the northern Adriatic Sea. Oceanologica Acta 13: 31-45.

7. Sinsabaugh RL, Findlay S (1995) Microbial production, enzyme activity, and carbon turnover in surface sediments of the Hudson River estuary. Microbial Ecology 30: 127-141.

8. Jorgensen SE, Bendoricchio G (2001) Fundamentals of Ecological Modelling. Oxford, UK: Elsevier Science

9. Holdren GC, Armstrong DE (1980) Factors affecting phosphorus release from intact lake sediment cores Environmental Science & Technology 14: 79-87.

10. Serruya C, Edelstein M, Pollingher U, Serruya S (1974) Lake Kinneret sediments: nutrient composition of the pore water and mud water exchanges. Limnology and Oceanography 19: 489-508.

11. Reynolds C (2006) The Ecology of Phytoplankton; Usher M, Suanders D, Peet R, Dobson A, editors. New York: Cambridge University Press.

12. Foy RH, Gibson CE, Smith RV (1976) The influence of daylength, light intensity, and temperature on the growth rates of planktonic blue-green algae. British Phycological Journal 11: 151-163.

13. Fogg GE (1949) Growth and heterocycst production in *Anabaena cylindrica Lemm II.* in relation to carbon and nitrogen metabolism Ann Botany London 13: 241-259.

14. Pollingher U, Berman T (1982) Relative contributions of net and nano-phytoplankton to primary production in Lake Kinneret (Israel) Archiv fur Hydrobiologie 96: 33-46.

15. Sandgren C (1988) Growth and Reproductive Strategies of Freshwater Phytoplankton; Sandgren C, editor: Cambridge University Press. v, 442 p. p.

16. Butterwick C, Heaney SI, Talling JF (2005) Diversity in the influence of temperature on the growth rates of freshwater algae, and its ecological relevance. Freshwater Biology 50: 291-300.

17. Sandgren (1995) Chrysophyte Algae: Ecology, Phylogeny, and Development: Cambridge University Press. xiv, 399 p. p.

18. Healey FP, Hendzel LL (1979) Indicators of phosphorus and nitrogen deficiency in 5 algae in cultures. Journal of the Fisheries Research Board of Canada 36: 1364-1369.

19. Dugdale VA, Dugdale RC (1962) Nitrogen metabolism in lakes. II. role of nitrogen fixation in sanctuary Lake Pennsylvania. Limnology and Oceanography 7: 170-177.

20. Imai H, Chang KH, Kusaba M, Nakano S (2009) Temperature-dependent dominance of *Microcystis* (Cyanophyceae) species: *M. aeruginosa* and *M. wesenbergii*. Journal of Plankton Research 31: 171-178.

21. Ukeles R (1961) The Effect of Temperature on the Growth and Survival of Several Marine Algal Species. Biological Bulletin 120: 255-264.

22. Robson BJ, Hamilton DP (2004) Three-dimensional modelling of a *Microcystis* bloom event in the Swan River estuary, Western Australia. Ecological Modelling 174: 203-222.

23. Gophen M (1976) Temperature effect on lifespan, metabolism, and development time of *Mesocyclops-leuckarti*. Oecologia 25: 271-277.

24. Demott WR (1982) Feeding selectivities and relative ingestion rates of Daphnia and Bosmina. Limnology and Oceanography 27: 518-527.

25. Gophen M (1976) Temperature-dependence of food-intake, ammonia excretion and respiration in Ceriodaphnia-reticulata (Lake Kinneret, Israel) Freshwater Biology 6: 451-455.

26. Lampert W (1986) Response of the respiratory rate of *Daphnia-magna* to changing food conditions Oecologia 70: 495-501.

27. Urabe J, Watanabe Y (1990) Influence of food density on respiration rate of 2 crustacean plankters, *Daphnia galeata* and *Bosmina longirostris*. Oecologia 82: 362-368.

28. Galkovskaja GA (1987) Planktonic rotifers and temperature Hydrobiologia 147: 307-317.

29. Bertilsson J, Berzins B, Pejler B (1995) The occurrence of limnic microcrustaceans in relation to temperature and oxygen Hydrobiologia 299: 163-167.

30. Landry M, Hassett R (1985) Time scales in behavioral, biochemical, and energetic adaptations to food-limiting conditions. Archiv fur Hydrobiologie: 209-211.

31. Haney J, Trout M (1985) Size selective grazing by zooplankton in Lake Titicaca. Archiv fuer Hydrobiologie: 147-160.

32. Stemberger RS, Gilbert JJ (1985) Body size, food concentration, and population growth in planktonic rotifers. Ecology 66: 1151-1159.

33. Andersen T, Hessen DO (1991) Carbon, nitrogen and phosphorus content of freshwater zooplankton. Limnology and Oceanography 36: 807-814.

34. Sterner RW, Hessen DO (1994) Algal nutrient limitation and the nutrition of aquatic herbivores. Annual Review of Ecology and Systematics 25: 1-29.

35. Dobberfuhl DR, Elser JJ (2000) Elemental stoichiometry of lower food web components in arctic and temperate lakes. Journal of Plankton Research 22: 1341-1354.

36. Jensen TC, Verschoor AM (2004) Effects of food quality on life history of the rotifer *Brachionus calycifloru*s Pallas. Freshwater Biology 49: 1138-1151.

37. Gophen M, Azoulay B (2002) The trophic status of zooplankton communities in Lake Kinneret (Israel). Verh Internat Verein Limnol 28: 836-839.

38. Lampert W (2007) Limnoecology: Oxford University Press Inc. ix, 324 p. p.

39. Lampert W (1974) Method for determining food selection by zooplankton Limnology and Oceanography 19: 995-998.

40. Hadas O, Malinsky-Rushansky N, Pinkas R, Cappenberg TE (1998) Grazing on autotrophic and heterotrophic picoplankton by ciliates isolated from Lake Kinneret, Israel. Journal of Plankton Research 20: 1435-1448.

41. Scavia D, Lang GA, Kitchell JF (1988) Dynamics of Lake Michigan plankton- A model evaluation of nutrient loading, competition, and predation Canadian Journal of Fisheries and Aquatic Sciences 45: 165-177.

42. Hamels I, Mussche H, Sabbe K, Muylaert K, Vyverman W (2004) Evidence for constant and highly specific active food selection by benthic ciliates in mixed diatoms assemblages. Limnology and Oceanography 49: 58-68.

43. Madoni P, Berman T, Hadas O, Pinkas R (1990) Food selection and growth of the planktonic ciliate *Coleps-hirtus* isolated from a monomictic subtropical lake Journal of Plankton Research 12: 735-741.

1. Gophen M (1976) Temperature effect on lifespan, metabolism, and development time o*f Mesocyclops-leuckar*ti. Oecologia 25: 271-277. [↑](#endnote-ref-2)
2. Demott WR (1982) Feeding selectivities and relative ingestion rates of Daphnia and Bosmina. Limnology and Oceanography 27: 518-527. [↑](#endnote-ref-3)
3. Gophen M (1976) Temperature-dependence of food-intake, ammonia excretion and respiration i*n Ceriodaphnia-reticula*ta (Lake Kinneret, Israel) Freshwater Biology 6: 451-455. [↑](#endnote-ref-4)
4. Lampert W (1986) Response of the respiratory rate o*f Daphnia-mag*na to changing food conditions Oecologia 70: 495-501. [↑](#endnote-ref-5)
5. Urabe J, Watanabe Y (1990) Influence of food density on respiration rate of 2 crustacean plankters*, Daphnia galea*ta an*d Bosmina longirostr*is. Oecologia 82: 362-368. [↑](#endnote-ref-6)
6. Galkovskaja GA (1987) Planktonic rotifers and temperature Hydrobiologia 147: 307-317. [↑](#endnote-ref-7)
7. Bertilsson J, Berzins B, Pejler B (1995) The occurrence of limnic microcrustaceans in relation to temperature and oxygen Hydrobiologia 299: 163-167. [↑](#endnote-ref-8)
8. Landry M, Hassett R (1985) Time scales in behavioral, biochemical, and energetic adaptations to food-limiting conditions. Archiv fur Hydrobiologie: 209-211. [↑](#endnote-ref-9)
9. Haney J, Trout M (1985) Size selective grazing by zooplankton in Lake Titicaca. Archiv fuer Hydrobiologie: 147-160. [↑](#endnote-ref-10)
10. Stemberger RS, Gilbert JJ (1985) Body size, food concentration, and population growth in planktonic rotifers. Ecology 66: 1151-1159. [↑](#endnote-ref-11)
11. Andersen T, Hessen DO (1991) Carbon, nitrogen and phosphorus content of freshwater zooplankton. Limnology and Oceanography 36: 807-814. [↑](#endnote-ref-12)
12. Sterner RW, Hessen DO (1994) Algal nutrient limitation and the nutrition of aquatic herbivores. Annual Review of Ecology and Systematics 25: 1-29. [↑](#endnote-ref-13)
13. Dobberfuhl DR, Elser JJ (2000) Elemental stoichiometry of lower food web components in arctic and temperate lakes. Journal of Plankton Research 22: 1341-1354. [↑](#endnote-ref-14)
14. Jensen TC, Verschoor AM (2004) Effects of food quality on life history of the rotife*r Brachionus calyciflo*ru*s Pall*as. Freshwater Biology 49: 1138-1151. [↑](#endnote-ref-15)
15. Gophen M, Azoulay B (2002) The trophic status of zooplankton communities in Lake Kinneret (Israel). Verh Internat Verein Limnol 28: 836-839. [↑](#endnote-ref-16)
16. Lampert W (2007) Limnoecology: Oxford University Press Inc. ix, 324 p. p. [↑](#endnote-ref-17)
17. Lampert W (1974) Method for determining food selection by zooplankton Limnology and Oceanography 19: 995-998. [↑](#endnote-ref-18)
18. Hadas O, Malinsky-Rushansky N, Pinkas R, Cappenberg TE (1998) Grazing on autotrophic and heterotrophic picoplankton by ciliates isolated from Lake Kinneret, Israel. Journal of Plankton Research 20: 1435-1448. [↑](#endnote-ref-19)
19. Scavia D, Lang GA, Kitchell JF (1988) Dynamics of Lake Michigan plankton- A model evaluation of nutrient loading, competition, and predation Canadian Journal of Fisheries and Aquatic Sciences 45: 165-177. [↑](#endnote-ref-20)
20. Hamels I, Mussche H, Sabbe K, Muylaert K, Vyverman W (2004) Evidence for constant and highly specific active food selection by benthic ciliates in mixed diatoms assemblages. Limnology and Oceanography 49: 58-68. [↑](#endnote-ref-21)
21. Madoni P, Berman T, Hadas O, Pinkas R (1990) Food selection and growth of the planktonic ciliat*e Coleps-hirt*us isolated from a monomictic subtropical lake Journal of Plankton Research 12: 735-741. [↑](#endnote-ref-22)
